# P300 response modulation reflects breaches of non-probabilistic expectations

**DOI:** 10.1038/s41598-020-67275-0

**Published:** 2020-06-24

**Authors:** D. Valakos, G. d’Avossa, D. Mylonas, J. Butler, C. Klein, N. Smyrnis

**Affiliations:** 1 Laboratory of Cognitive Neuroscience and Sensorimotor Control, University Mental Health, Neurosciences and Precision Medicine Research Institute “COSTAS STEFANIS”, Athens, Greece; 2 Department of Psychology, Bangor University, Bangor, UK; 3 Department of Psychiatry, National and Kapodistrian University of Athens, Medical School, Eginition Hospital, Athens, Greece; 4 MGH/HST Athinoula A. Martinos Center for Biomedical Imaging, Massachusetts General Hospital, Boston, MA USA; 5 Department of Psychiatry, Harvard Medical School, Boston, MA USA; 6 School of Psychology, Faculty of Health and Wellbeing, University of Sunderland, Sunderland, UK; 7 Department of Child and Adolescent Psychiatry, University of Freiburg, Freiburg, Germany; 8 Department of Child and Adolescent Psychiatry, Medical Faculty, University of Cologne, Cologne, Germany; 9 Faculty of Biology, National and Kapodistrian University of Athens, Panepistimioupoli Zografou, Athens, Greece

**Keywords:** Cognitive neuroscience, Attention

## Abstract

In oddball paradigms, infrequent stimuli elicit larger P300 event related potentials (ERPs) than frequent ones. One hypothesis is that P300 modulations reflect the degree of “surprise” associated with unexpected stimuli. That is the P300 represents how unlikely the stimulus is and this signal is then used to update the observer’s expectations. It could be hypothesized that P300 is modulated by any factor affecting an observer’s expectations, not only target probability. Alternatively, the P300 may reflect an evaluative process engaged whenever a discrepancy between task context and sensory inputs arises, irrespective of the latter probability. In previous ERP studies, stimulus probability was often the only determinant of task set confounding the effects of stimulus probability and set stimulus discrepancy. In this study, we used a speeded luminance detection task. The target was preceded by a central cue that predicted its location. The probability that the target was valid, i.e. would appear at the cued location was manipulated by varying the reliability of the cue. Reaction times were modulated by probabilistic expectations based on cue reliability and target validity while P300 was affected by target validity only. We conclude that increased P300 amplitude reflects primarily breaches of non-probabilistic expectations, rather than target probability.

## Introduction

Event Related Potentials (ERPs) are electroencephalographic signals time-locked to environmental and/or internal events. The most studied ERP is the P300^1^, a positive component with centro-parietal scalp topography that follows sensory ERPs about 300 ms after stimulus onset. Stimulus frequency modulates P300 amplitude since larger responses are elicited by infrequent (oddball) targets, randomly interspersed among high frequency (standard) stimuli^2^. Familiarization with the probability distribution of several imperative targets, in choice reaction time tasks, produces trial by trial variations in P300 amplitude reflecting the surprise associated with each target, which changes as the participants learn the targets’ probability distribution^3^. These results have suggested that P300 amplitude tracks target probability, increasing when surprising, unexpected stimuli occur^4–6^. Accordingly, the P300 represents an error signal, which learning mechanisms aim to minimize based on the observed distribution of events. A more general and unifying hypothesis suggests that P300 modulation might reflect an updating process initiated when a discrepancy arises between the current stimulus and the task context and is not restricted to a mechanism tracking target probability^4–7^.

Some of the evidence for this later proposal comes from cued detection tasks, where a central cue indicates the likely location of an upcoming target which may appear at the indicated location (valid target) or elsewhere (invalid target). This task provides the opportunity to study spatial orienting, while minimizing sensory, motor and decision confounds^8^. It has been suggested that attention and expectations have distinct roles in modulating sensory responses^9,10^. However, while it is still unclear whether expectations improve accumulation of sensory data or simply bias decision processes^11,12^, the proposal that central cues affect spatial orienting has not been challenged. Thus, the difference between reaction times (RT) elicited by valid and invalid targets, namely the validity effect, has been interpreted as reflecting both gains obtained by anticipatory allocation of attention to the target location and costs incurred by the need to reorient attention, when the target occupies a location different form the cued one. Crucially, the use of highly visible targets precludes an alternative interpretation, namely that the effects of target validity reflect changes in the rate of accumulation of sensory information^8,13^. The differential response to valid and invalid targets is referred to as the validity effect. Behaviorally, valid targets produce faster Reaction Times (RTs) than invalid ones. The P300 amplitude is also modulated by target validity, being larger following invalid than valid targets^14,15^. While previous studies did not always separate the effects of target validity and target probability^5,7^ recently Arjona *et al*.^16^ showed that the P300 evoked by monaural auditory targets was also affected by the target validity and the previous cue reliability, suggesting that target probability modulates the P300. In that study, the cue predicted both the ear where the sound was going to be presented as well as the hand used to respond, therefore whether the P300 modulation was due to attentional processes, motor processes or both was left unsettled. BOLD imaging studies have indeed indicated that breeches of attentional expectations and motor preparation are associated with distinct cortical responses^17^.

More broadly, fMRI studies have uncovered the cortical network involved in attentional orienting, following the cue, and reorienting, following unexpected, but task relevant targets^18^. The right Temporal Parietal Junction (rTPJ), a region also thought to be a major generator of the P300^19^, is prominently involved in reorienting to task relevant stimuli, when these appear outside the current focus of attention. Target validity and cue reliability were both found to affect the target evoked BOLD responses in the rTPJ, which shows larger responses to invalid targets preceded by high than low reliability cues, suggesting that unexpected shifts of attention produce greater activations than expected ones, whether the cue reliability is instructed explicitly^20^ or learnt implicitly^21^. In contrast, Shulman *et al*.^22^ found that BOLD responses evoked by a cue, which appeared either at the attended location and instructed participants to keep attending it or in the opposite visual hemifield and instructed participants to shift attention, were larger following shift than stay cues. However, cue evoked responses in the rTPJ were not further modulated by the probability that the cue would instruct a stay vs. a shift of attention, in contrast to most other cortical and subcortical regions where larger BOLD response followed low probability, shift cues. Doricchi *et al*.^23^ also found that target evoked responses in rTPJ were modulated only by the validity of the target, but not the reliability of the preceding cue. These findings suggest that target validity and target probability might, to some extent, be processed independently in cortical networks.

**Figure 1:**
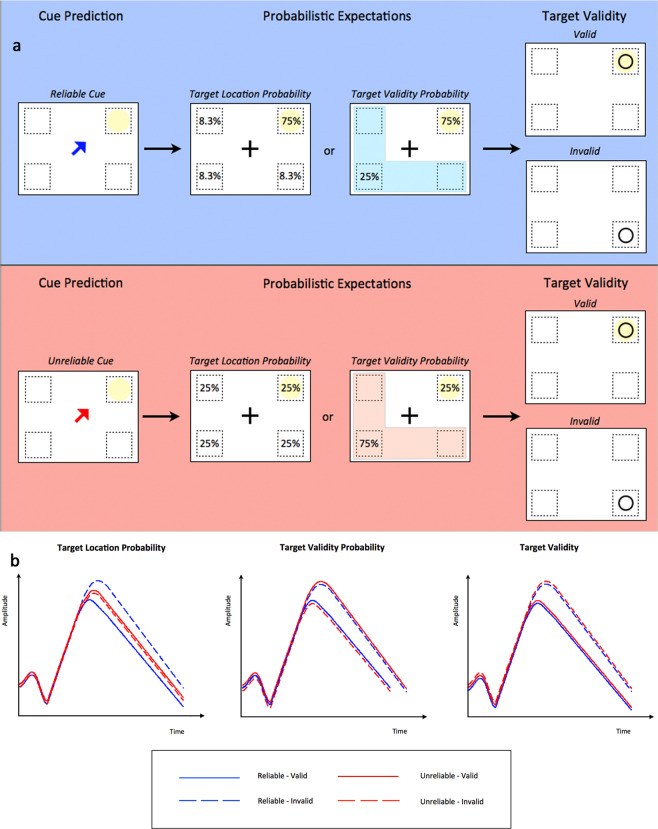
(a) Representation of the spatial cueing task. **Blue panel:** a reliable blue cue indicates the location of the upcoming target in 75% of trials. Therefore, the probability that the target appears at the indicated location equals with 0.75 (valid prediction), while the probability that the target appears at a location different from the indicated one is 0.25 (invalid prediction). As a result, the probability that the target appears in each of the invalid target locations equals to 0.083. **Red panel**: an unreliable red cue indicates the location of the upcoming target in 25% of trials. Therefore, the probability that the target appears at the indicated location equals with 0.25 (valid prediction), while the probability that the target appears at a location different from the indicated one is 0.75 (invalid prediction). As a result, the probability that the target appears in each of the invalid target locations equals to 0.25. **b)** Prediction of P300 response modulation according to target location probability (left panel), target validity probability (central panel) and target validity (right panel) hypotheses. In the target location probability the P300 response is dependent on the probability of target location being smaller for reliable-valid trials (P = 0.75), intermediate for unreliable-valid and unreliable-invalid trials which share the same target location probability (P = 0.25) and larger for reliable–invalid trials (P = 0.083). For the target validity probability hypothesis, P300 should be smaller for reliable-valid trials and unreliable-invalid trials (P = 0.75) (standard trials) and larger for reliable-invalid and unreliable-valid trials (P = 0.25) (oddball trials). Finally for the target validity hypothesis, P300 should be larger for invalid compared to valid trials.

**Figure 2:**
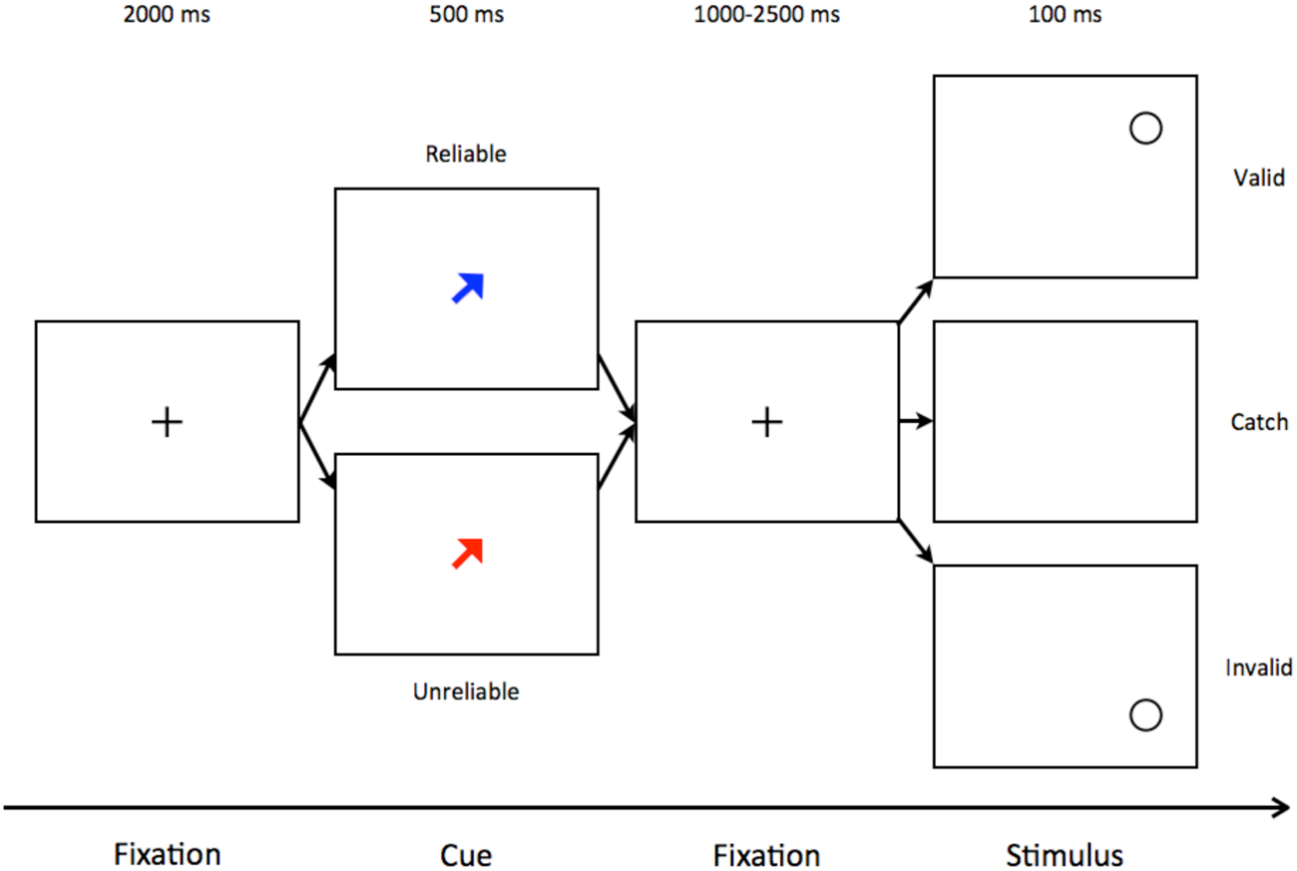
Diagrammatic representation of the task. A black cross was used as a fixation point for 2000ms (fixation period), which was replaced by a central arrow (cue) indicating one of the four possible quadrants where the target could appear: up-right, up-left, down-right, down-left. The cue remained visible for 500 ms (cue period) and was then replaced by the central fixation cross that remained visible for a variable non-aging fore period lasting 1000–2500 ms (pre-stimulus period). Then a black circle appeared (target) for 100 ms either at the cued location or one of the other three possible locations. In 10% of trials no target stimulus appeared (catch trials). Finally, the target was replaced by the central fixation cross initiating the next trial. Cues could be either reliable (the arrow was coloured blue) or unreliable (the arrow was coloured red) and participants were informed about the mapping between cue colour and reliability, before the onset of the experiment.

In the present study, we utilized a version of Posner’s cueing paradigm^8^. We manipulated the probability that a luminance target would be valid or invalid by varying the reliability of the preceding cue over two levels (Fig. 1a): when the cue was reliable (blue color), the target appeared at the cued location in 75% of trials (valid target) and in one of the other three locations in 25% of trials (invalid target); when the cue was unreliable (red color) the target appeared at the cue location in 25% of trials (valid target) and in one of the other three locations in 75% of trials (invalid target). Three alternative hypotheses were tested (Fig. 1b, Supplementary Table 1). The first hypothesis was that P300 amplitude modulations reflect breaches in the expectation of target location based on the reliability of the cue (herein defined as target location probability hypothesis). Thus, P300 amplitude should be smallest when a valid target follows a reliable cue (probability of the target appearing at the cued location: 0.75) and largest when an invalid target follows a reliable cue (probability of the target appearing at a location different from the cued: 0.083). The P300 amplitude should not differ when the cue is unreliable for both valid and invalid target locations (probability of the target appearing at the cued location or any other location: 0.25). Also since the target location probability for unreliable cues is three fold larger than the probability of an invalid target after a reliable cue and three fold smaller than the probability of a valid target after a reliable cue, the P300 response for all unreliable targets should be intermediate between the P300 response for invalid and valid reliable cues (Fig. 1b, left panel). The second hypothesis was that P300 reflects a probabilistic prediction of target’s validity (target validity probability). Accordingly, the P300 response should be larger when an invalid target was preceded by a reliable cue and a valid target preceded by an unreliable cue both having a probability of 25% (oddball condition). The P300 response should be smaller when a valid target was preceded by reliable cue and an invalid target preceded by an unreliable cue both having a probability of 75% (standard condition) (Fig. 1b, central panel). The third hypothesis was that P300 reflects target validity. Thus, P300 response should be larger for invalid compared to valid target irrespective of cue reliability (Fig. 1b, right panel). The timeline of the task is described in detail at the Materials and Methods section (Fig. 2).

Two experiments were performed. In the first one, trials containing both reliable and unreliable cues were randomly intermixed (mixed design) while in the second, the cue reliability was blocked. The latter design yielded larger behavioral effects of cue reliability and therefore provided a more stringent test of the hypotheses listed above.

## Results

### Behavioral Results

#### Experiment 1 (Mixed Design)

**Figure 3:**
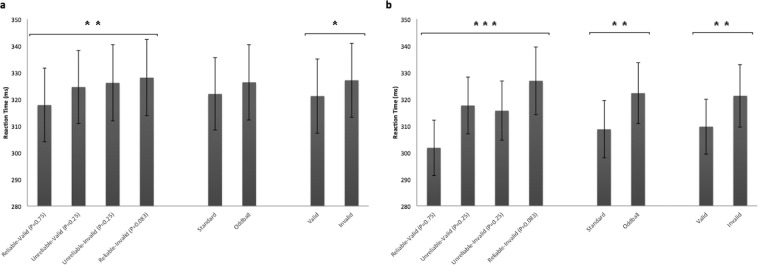
Bar graphs depicting mean RTs for target location probability, target validity probability and target validity hypotheses in the mixed design (**a**) and block (**b**) design experiments. Error bars represent standard errors of the means. *: p < 0.05, **: p < 0.01, ***: p < 0.001.

Participants responded significantly faster to valid (Mean = 321.21 ms, SE = 13.6 ms) than invalid (Mean = 327.13 ms SE = 14.21 ms) targets (F_1,12_ = 6.98, *p* = 0.02, partial η^2^ = 0.37) (Fig. 3a, Supplementary Fig. 1a). Cue reliability (Mean for reliable = 322.97 ms, SE = 13.93 ms; Mean for unreliable = 325.37 ms, SE = 13.85 ms) (F_1,12_ = 1.82, *p* = 0.2, partial η^2^ = 0.13) and its interaction with target validity (Fig. 3a, Supplementary Fig. 1a) (F_1,12_ = 3.17, *p* = 0.1 partial η^2^ = 0.21) had no significant effect on RT. The effect of target location probability on RT was significant (F_1,12_ = 7.31, *p* = 0.02 partial η^2^ = 0.37) and RT was fastest for high probability target locations (0.75) slowest for low probability target locations (0.083) and intermediate for the 0.25 probability target locations where both types of trials (unreliable valid and unreliable invalid) resulted in similar RTs (Fig. 3a, Supplementary Fig. 1a). The effect of target validity probability on RT did not reach significance in the mixed design (F_1,12_ = 3.17, *p* = 0.1 partial η^2^ = 0.21) (Fig. 3a, Supplementary Fig. 1a).

### Experiment 2 (Blocked Design)

Participants responded significantly faster to valid (Mean = 309.66 ms, SE = 10.32 ms) than invalid targets (Mean = 321.22 ms, SE = 11.72 ms) (F_1,9_ = 15.94, *p* = 0.003 partial η^2^ = 0.64) (Fig. 3b, Supplementary Fig. 1b). Cue reliability had no significant effect on RTs (Mean for reliable = 314.28 ms, SE = 11.56 ms; Mean for unreliable = 316.60 ms, SE = 10.65 ms) (F_1,9_ = 0.59, *p* = 0.46, partial η^2^ = 0.06). However, there was a significant interaction between target validity and cue reliability for the block design (F_1,9_ = 15.91, *p* = 0.003, partial η^2^ = 0.64), since the validity effect was larger following reliable than unreliable cues (Fig. 3b, Supplementary Fig. 1b). The effect of target location probability on RT was significant (F_1,9_ = 21.59, *p* = 0.001, partial η^2^ = 0.7) showing the same variation of RT with target location probability as that observed in experiment 1 (Fig. 3b, Supplementary Fig. 1b). The effect of target validity probability on RT was significant in the block design with faster responses in the case of standard trials (F_1,9_ = 49.23, *p* = 0.00006, partial η^2^ = 0.64) (Fig. 3b, Supplementary Fig. 1b).

### ERP results

**Figure 4:**
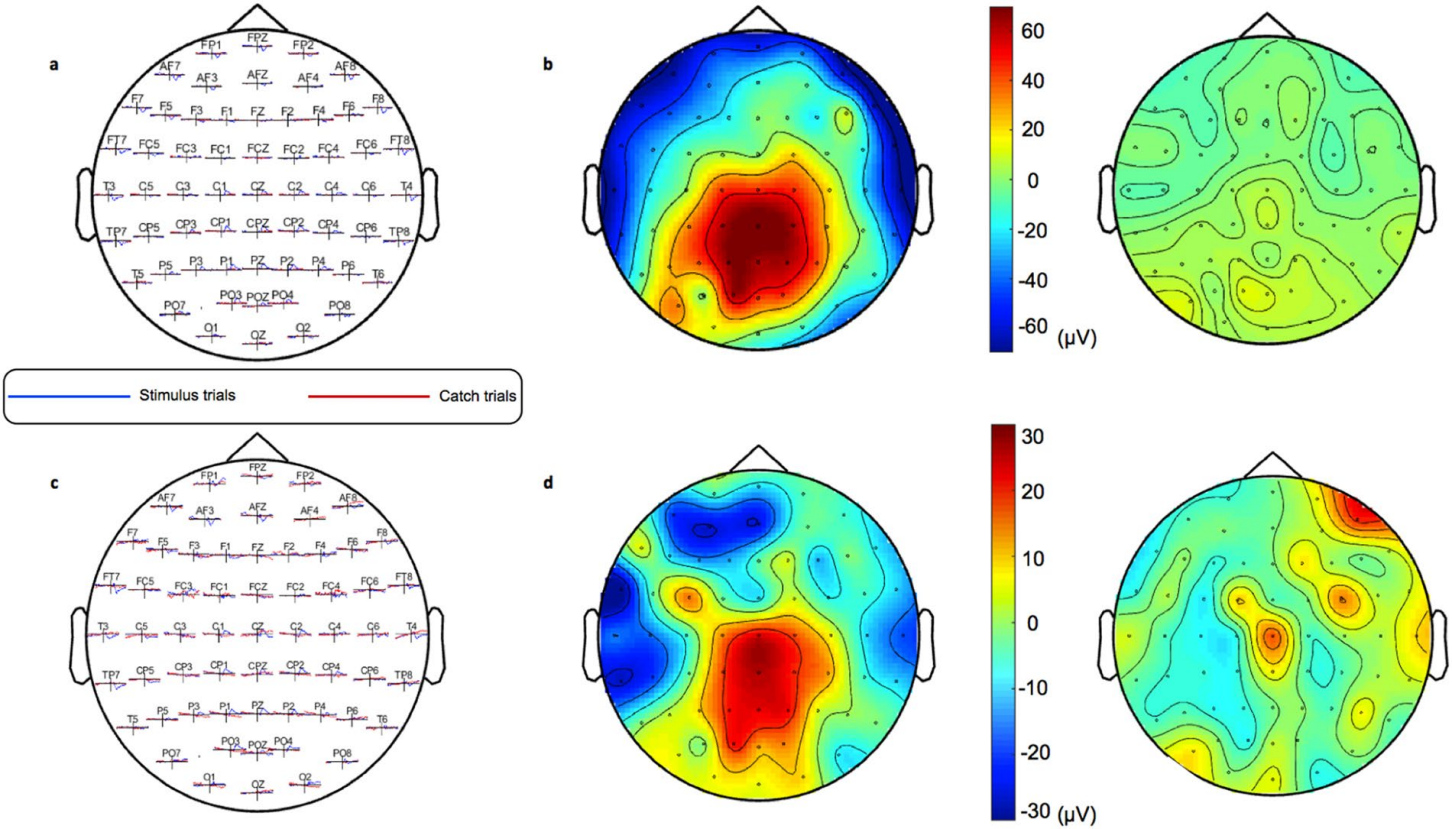
**a**. ERPs for all the recorded electrode sites for the stimulus trials (blue) and catch trial (red) in the mix design experiment. **b**. Heat-maps depicting the grand average ERP topography between 350 and 450 ms post stimulus for the stimulus trials (left) and the catch trials (right) in the mix design experiment. **c**. ERPs for all the recorded electrode sites for the stimulus trials (blue) and catch trial (red) in the block design experiment. **d**. Heat-maps depicting the grand average ERPs topography between 350 and 450 ms post stimulus for the stimulus trials (left) and the catch trials (right) in the block design experiment.

The initial analysis of the ERPs confirmed a significant activation at the 200–500 ms time window post stimulus in the stimulus presentation trials compared to the catch trials both in the mixed design (Fig. 4a) and the block design (Fig. 4c). This activation had a centro-parietal distribution over both hemispheres for the mixed (Fig. 4b) and the block design experiments (Fig. 4d). Thus, this ERP component had the spatiotemporal characteristics of a P3 potential response and particularly the P3b.

#### Experiment 1 (Mixed Design)

**Figure 5:**
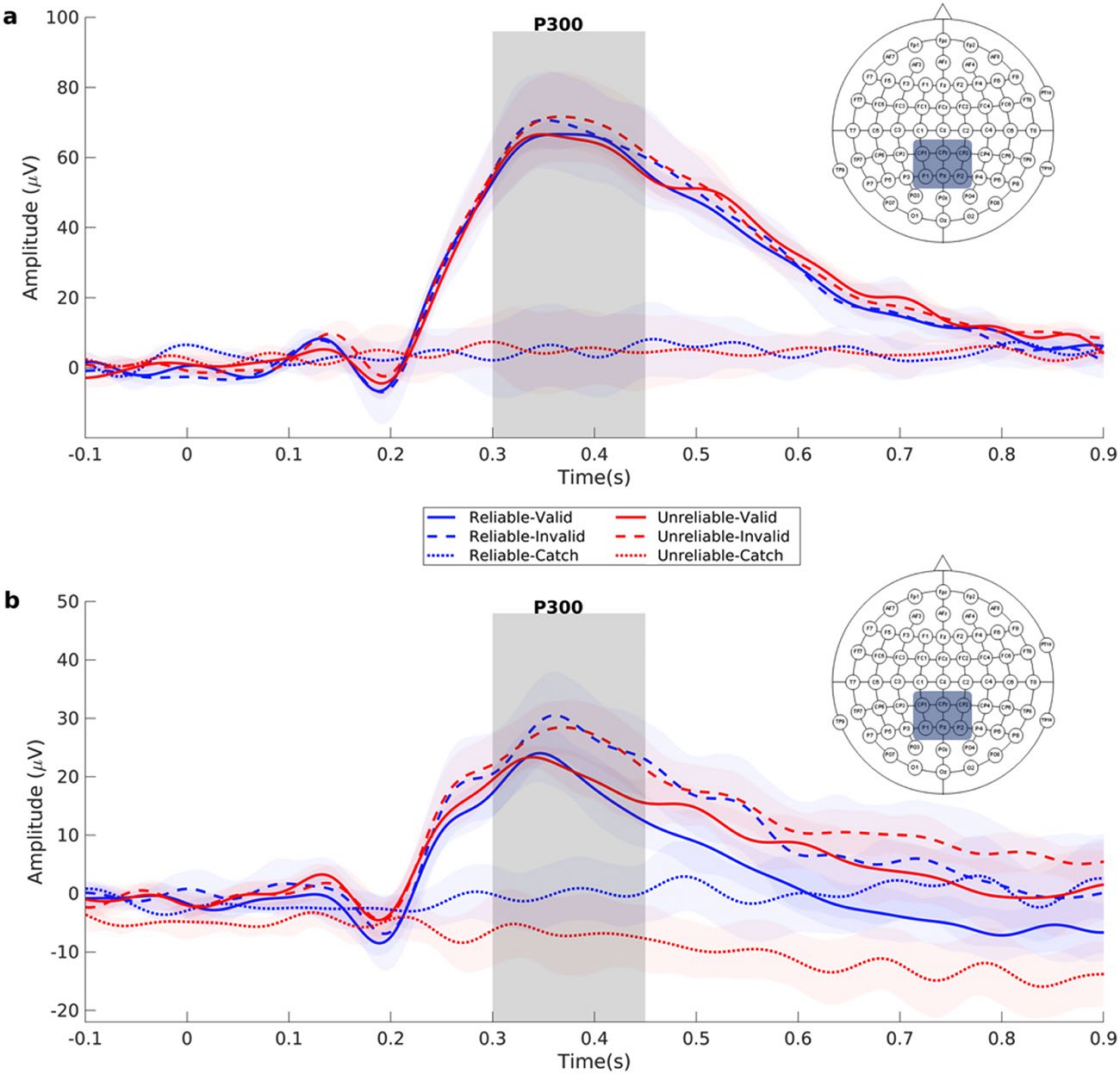
Grand average ERPs from the selected central-parietal electrodes (CP_1_, CP_z_, CP_2_, P_1_, P_z_, P_2_) for the mixed design (**a**) and blocked design (**b**) respectively. The mapping of each condition is represented in a frame below the waveforms. The shaded areas in each waveform depict the standard error of the mean. Invalid trials produce more robust P300 responses in both designs.

**Figure 6:**
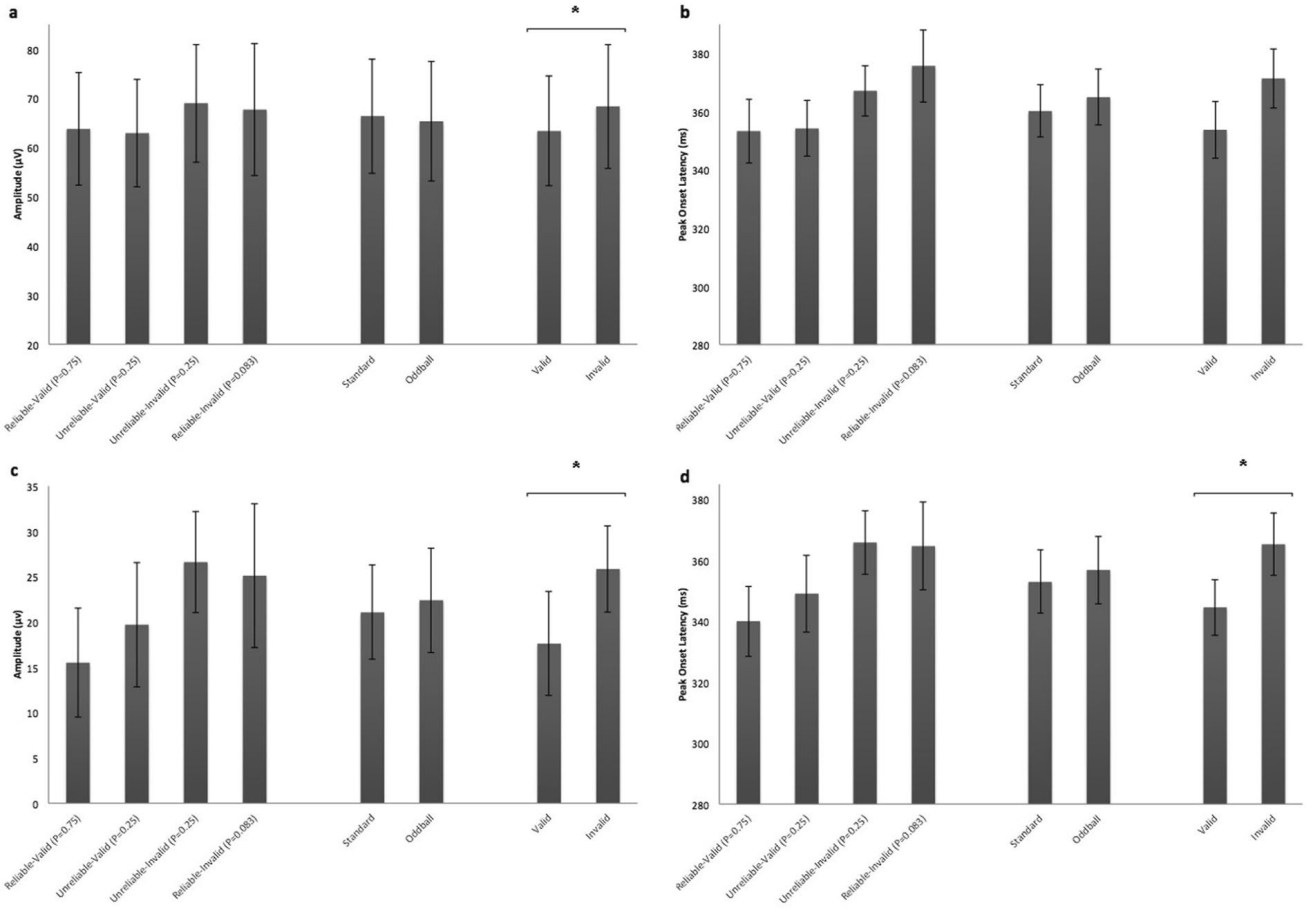
Graph bars depicting mean P300 amplitude and latency for target location probability, target validity probability and target validity hypotheses in the mixed design (**a** and **b** respectively) and in the blocked design (**c** and **d** respectively). Error bars represent standard errors of the means. *: p < 0.05.

Figure 5a shows the grand average ERP waveforms for the mixed design experiment respectively, measured over the centro-parietal electrodes that produce a robust P300 potential. Invalid trials produced more robust P300 responses compared to valid trials. Particularly, P300 amplitude was significantly larger following invalid compared to valid targets (F_1,12_ = 5.16, *p* = 0.04, partial η^2^ = 0.30) (Fig. 6a, Supplementary Fig. 2a) while there was no significant effect of cue reliability (Mean for reliable = 65.73μV, SE = 12.38μV; Mean for unreliable = 65.96μV, SE = 11.37μV) (F_1,12_ = 0.02, *p* = 0.9, partial η^2^ = 0.001) or interaction of cue reliability and target validity (Fig. 6a, Supplementary Fig. 2a) (F_1,12_ = 0.03, *p* = 0.6, partial η^2^ = 0.02). There was no effect of target location probability (F_1,12_ = 1.33, *p* = 0.27, partial η^2^ = 0.01) or validity probability (F_1,12_ = 0.31, *p* = 0.59, partial η^2^ = 0.02) on P300 amplitude (Fig. 6a, Supplementary Fig. 2a).

In order to provide further validation for the observed effects of reliability and validity on P300 amplitude we applied Bayesian factor analysis (see methods). The Bayes factor comparing the null/alternative hypothesis for the main effect of reliability was 3.6 (error 0.017) indicating ‘moderate’ evidence^24^ favoring the null hypothesis namely the absence of reliability effect for P300 amplitude. Similarly the Bays factor comparing the null/alternative hypothesis for the interaction of validity and reliability was 3.1 (error 0.007) indicating ‘moderate’ evidence for the null hypothesis, namely the absence of validity-reliability interaction for P300 amplitude. Finally, the Bayes factor for comparing the null/alternative hypothesis that the P300 amplitude was larger for invalid versus valid trials was 3.6 (error 0.001) again providing ‘moderate’ evidence in favor of the alternative hypothesis.

Peak P300 latency was longer for invalid compared to valid targets, an effect which did not reach significance (F_1,12_ = 3.64, *p* = 0.08, partial η^2^ = 0.23) (Fig. 6b, Supplementary Fig. 2b). Neither the effect of cue reliability (Mean for reliable = 364.57 ms, SE = 9.59 ms; Mean for unreliable = 360.71 ms, SE = 8.33 ms) (F_1,12_ = 1.77, *p* = 0.21, partial η^2^ = 0.13) nor the interaction of reliability and validity were significant (Fig. 6b, Supplementary Fig. 2b) (F_1,12_ = 0.67, *p* = 0.4, partial η^2^ = 0.05). There was no effect of target location probability (F_1,12_ = 2.99, *p* = 0.11, partial η^2^ = 0.2) or target validity probability (F_2,24_ = 2.68, *p* = 0.09, partial η^2^ = 0.18) (F_1,12_ = 0.67, *p* = 0.43, partial η^2^ = 0.05) on P300 latency (Fig. 6b, Supplementary Fig. 2b).

#### Experiment 2 (Blocked Design)

Figure 5b shows the grand average ERP waveforms for the block design experiment, estimated over the centro-parietal electrodes that produced a robust P300 potential. P300 amplitude was larger following invalid than valid targets (F_1,9_ = 5.26, *p* = 0.047, partial η^2^ = 0.37) (Fig. 6c, Supplementary Fig. 2c). Neither cue reliability (Mean for reliable = 20.30μV, SE = 5.93μV; Mean for unreliable = 23.15μV, SE = 5.95) (F_1,9_ = 0.19, *p* = 0.68, partial η^2^ = 0.20) nor the cue reliability by target validity interaction (F_1,9_ = 0.08, *p* = 0.79, partial η^2^ = 0.01) (Fig. 6c, Supplementary Fig. 2c) appreciably affected P300 amplitudes. There was no effect of target location probability (F_1,9_ = 0.68, *p* = 0.43, partial η^2^ = 0.07) nor target validity probability (F_1,9_ = 0.08, *p* = 0.79, partial η^2^ = 0.01) on P300 amplitude (Fig. 6c, Supplementary Fig. 2c).

Further validation for the observed effects of reliability and validity on P300 amplitude was provided by Bayesian factor analysis (see methods). The Bayes factor comparing the null/alternative hypothesis for the main effect of reliability was 3.0 (error 0.006) providing ‘moderate’ evidence in favor of the null hypothesis of absence of reliability effect on P300 amplitude. Similarly the Bays factor comparing the null/alternative hypothesis for the interaction of validity and reliability was 3.1 (error 0.017) providing ‘moderate’ evidence for the null hypothesis of absence of validity-reliability interaction on P300 amplitude. Finally the Bayes factor comparing the null/alternative hypothesis that the P300 amplitude was larger for invalid versus valid trials was 3.5 (error 0.001) providing again ‘moderate’ evidence for the alternative hypothesis.

Peak P300 latency was longer for invalid compared to valid targets (Mean = 344.54 ms, SE = 9.15 ms) (F_1,9_ = 8.29, *p* = 0.02, partial η^2^ = 0.48) (Fig. 6d, Supplementary Fig. 2d). Neither the effect of cue reliability (Mean for reliable = 352.34 ms, SE = 11.01 ms; Mean for unreliable = 357.45 ms, SE = 9.59 ms) (F_1,9_ = 0.26, *p* = 0.62, partial η^2^ = 0.03) nor the interaction of reliability and validity were significant (F_1,9_ = 0.11, *p* = 0.7, partial η^2^ = 0.11) (Fig. 6d, Supplementary Fig. 2d). There was no effect of target location probability (F_1,9_ = 1.82, *p* = 0.17, partial η^2^ = 0.17) or target validity probability (F_1,9_ = 0.11, *p* = 0.74, partial η^2^ = 0.01) on P300 latency (Fig. 6d, Supplementary Fig. 2d).

## Discussion

The P300 has been suggested to reflect the mismatch between the observer’s expectations and sensory inputs, since rare/surprising stimuli produce larger P300s compared to frequent/expected ones^5^. The P300 elicited by infrequent stimuli has been named the P3b response and is characterized by a centro-parietal scalp distribution while the P300 elicited by novel stimuli has a more frontal distribution and has been named the P3a response^4,31^. Infrequent stimuli are commonly used to elicit P300 responses and a number of studies concluded that the P300 signal reflects estimates of the target probability, which may inform the learning of the probability distribution of behaviorally relevant events^3^^,^ A more general hypothesis of the functional significance of the P300 is that it reflects processes evaluating the importance of stimuli that breach the current task context^4,5^.

A robust modulation of the P300 response and more specifically the P3b response has also been observed in spatial cueing tasks where invalid targets produce larger P300 responses than valid targets^8^, leading to the suggestion that validity effects reflect matching of expectations and sensory inputs^19^. Accordingly, the P300 would represent an error signal used to update estimates of the cue reliability^20^ or equivalently, the magnitude of the adjustment made to estimates of the cue reliability^21^. While most previous studies did not separate the effects of target validity and target probability, Arjona *et al*.^16^ compared the P300 evoked by invalid and valid auditory targets, which followed a spatial cue whose reliability varied from block to block. They found that the validity effect was modulated by the reliability of the cue, being largest for invalid targets preceded by reliable cues. An important difference between present study and that of Arjona *et al*.^16^ was that in that study the cue also instructed specific motor preparation (responding using either the right or left hand). Thus the P300 response modulations in that study could be assessing the adequacy of the cue predicted sensory-motor representation and not the cue-target sensory representation as in present report. Using the same paradigm in which the cue predicted both the stimulus and the specific motor response these authors further showed that trial by trial P300 modulations were related to an updating of the trial by trial probabilistic relationship between cues and targets based on previous cue-target outcomes^25,26^. Lasaponara *et al*.^27^ also examined the effects of cue reliability on detection latencies and ERPs. They found that cue reliability affects detection latencies to invalid targets only. They also reported effects of cue reliability and target validity on early ERP components, but not the P300. The conclusion drawn by the authors of that study was that mechanisms highlighting attended locations are separate from those inhibiting unattended ones. However, the findings of that study are confounded by the fact that manipulation of cue reliability changed more the log likelihood of invalid than valid targets. In the current study, we examined the effects of cue reliability and target validity on the P300 in a simple reaction detection task. The design also equalized log likelihood changes, produced by changes in cue reliability, for valid and invalid targets. We found that target validity modulated the P300 amplitude. However, the reliability of the preceding cue did not. Interestingly, RTs showed greater validity effects following reliable than unreliable cues, this difference being significant in the blocked design. Therefore, our electrophysiological results suggest that validity effects can arise separately and independently of cue reliability, in keeping with the idea that attention and expectancy are distinct processes^22^.

Remarkably, we observed a large P300 amplitude difference in the mixed design compared to the blocked design. Since this was an unexpected finding in this study, the design did not allow us to further investigate the P300 difference between the block and mixed design that would have to be systematically varied in a within subject design. An obvious suggestion is that this difference reflects the cognitive demands posed by the two task designs: the mixed design required participants to keep track of the cue reliability trial by trial, while the blocked design required them to do so once at the beginning of the block. Unfortunately, the existing literature does not clearly support the notion that increased task complexity predicts greater P300 amplitude^4,28^. Nevertheless, one possibility is that the P300 amplitude also reflects processes which reconfigure the task-set in preparation for the next cue. These processes would not only have to reset orienting signals, but also signals tracking the previous trial cue reliability in the mixed design, hence the larger neural response and P300 amplitude in the mixed than block design task.

More importantly, these findings are inconsistent with the hypothesis that target probability is the main modulator of the P300 amplitude. If target probability was the main variable affecting P300 amplitude then no validity effect or a reversal of the validity effect should have been observed after unreliable cues. Instead we found P300 validity effects following both reliable and unreliable cues, in keeping with previous behavioral^23,29^ as well as physiological data^10,14,17^. Target location probability had an effect on reaction times in both tasks and target validity probability had an effect on RT in the blocked design, suggesting that there are processes which track probabilistic expectations engendered by the cue on the basis of its reliability. However, the effects of cue reliability on the P300 were negligible compared to those of target validity. fMRI findings support the existence of neural mechanisms which are engaged by reorienting independently of its probability^22^.

In conclusion, our results provide evidence against the hypothesis that P300 (P3b component) primarily represents target probability, contrary to previous conclusions. Rather, the current study showed that the main factor modulating P300 is the discrepancy between the expected target location, instructed by the cue and the actual target location, which requires the reorientation of attention to a previously ignored location. The Posner paradigm, where the effects of cue are not fully accounted by its reliability, provides a convenient tool to dissociate the neural processes associated with specifying, respectively, the task context and probabilistic expectancies.

## Methods

### Participants

27 healthy participants participated in the study (10 women, 17 men, mean age=22.3years, SD = 1.85years). 17 participants performed the mixed design experiment and 10 new participants performed the block design experiment (see below). The two experiments were performed independently (initially the mixed design followed by the blocked design). All participants were right-handed and they were screened for a negative history of neurological or psychiatric disorders as well as any medication or psychoactive substance use. Experiments were approved by the ethics committee of University Mental Health Research Institute (U.M.H.R.I.) in accordance with the relevant guidelines and regulations, and participants signed a written informed consent.

### Task Procedure

Participants sat comfortably in front of a 22inch computer monitor (LG, IPSLED 22MP65) placed on a table at a distance of approximately 50 cm from them. The participants kept the right index finger on a response button console (Cedrus model Lumina). Each trial began with the appearance of black cross (2 cm ×2 cm) at the centre of a 22inches computer screen which served as fixation (Fig. 2). After 2000ms (fixation period) the cross was replaced by a central arrow cue (2 cm ×2 cm) indicating one of the four possible quadrants where the target could appear: up-right, up-left, down-right, down-left at 9 degrees of visual angle from the fixation point at the centre of the screen. The arrow cue remained visible for 500 ms (cue period) and was then replaced by the central fixation cross that remained visible for a variable non-aging fore-period lasting 1000–2500 ms (pre-stimulus period). Then a target (a 2 cm ×2 cm black circle appeared for 100 ms either at the cued location or one of the other three possible locations. In 10% of trials no target stimulus appeared (catch trials). Finally, the target was replaced by the central fixation cross initiating the next trial. Participants were asked to respond by pushing a single button with their right index finger as quickly as possible after the appearance of the target stimulus at any one of the four target locations. There was no intention to differentiate the response towards the four target locations. They were also instructed to maintain fixation at the centre of the screen and avoid making saccades.

Arrow cues could be either reliable (the arrow was coloured blue) or unreliable (the arrow was coloured red). The prediction of reliable cues was valid in 75% of the respective trials and invalid in 25% of them, while the prediction of unreliable cues was valid in 25% of the respective trials and invalid in 75% of them. Participants were informed about the mapping between cue colour and reliability before the onset of the experiment.

Two experimental designs were used (Supplementary Table 1). In the *mixed design*, subjects performed three blocks of 122 trials each. Each block included 61 trials with reliable and 61 trials with unreliable cues that were randomly mixed. In 36 trials with reliable cues, the target appeared at the cued location while in 12 trials it appeared at one of the un-cued locations. In 13 catch trials no target appeared. In 36 trials of those involving unreliable cues (red coloured cue), the target appeared at one of the un-cued locations, in 12 trials it appeared at the cued location, and 13 were catch trials. The order of the trials was randomized except that no two catch trials could appear sequentially. In the *blocked design*, two separate blocks of 61 trials were run consecutively, one containing only reliable cues and one containing only unreliable cues for a total of 6 blocks. The order of blocks was counterbalanced across participants. Stimuli presentation was implemented using a custom coded script in E-Prime version 1.2 software (Psychology Tools Software, U.S.A.).

### Behavioural data analysis

Reaction times (RTs) were lost in 3 subjects in the mixed design only, due to a hardware problem.

RTs whose latency varied between 150 and 600 ms were included in the final analysis. This resulted in the removal of 1.4% of the trials for the mixed design, and 1.2% of trials for the block design. Notably, the percentage of erroneous responses in catch trials, namely trials without target to which participants responded, correspond to 0.3% for the mixed design and 0% for the block design. For each subject the mean RT was computed for each trial type (reliable valid, reliable invalid, unreliable valid, unreliable invalid). A repeated measures, two way ANOVA was used to assess the effects of target validity (valid vs invalid) and cue reliability (75% vs 25%) as well as their interaction. To test the specific hypothesis (first hypothesis presented in the introduction) that RT was related to the predicted target probability we used a planned comparison of means. The vector contrasts had different signs for valid reliable and invalid reliable trials, while the unreliable trials were collapsed in one contrast. Another planned comparison was used to test the cue match probability effect with two opposite contrasts modeling the RT difference between cue reliable/valid target plus unreliable cue/invalid target trials versus reliable cue/invalid target plus unreliable cue/valid target trials. The General Linear Model module of the STATISTICA 10.0 software (StatSoft Inc. 1984–2011) was used for statistical analysis.

### EEG recording

EEG was recorded using the ISO-1064CE and CONTROL-1164 Braintronics system (Almere, The Netherlands) from 61 scalp sites according to an extended version of 10–20 System, using a cap with Ag/AgCl passive type electrodes (Micromed S.P.A., Treviso, Italy). For impedance reduction both abrasive type (Νeuprep, Spes Medica s.r.l., Genova, Italy), followed by electrolyte type (Νeurgel, Spes Medica s.r.l., Genova, Italy) gel was used. Impedance of electrodes was maintained under 5 KΩ. The EEG cap had integrated reference and ground electrodes, the former between CPz and Pz electrode sites and the latter between Fz and AFz electrode sites. The EEG signals were recorded using a 10 s time constant and a 100 Hz analogue low pass filter. An inbuilt analogue notch filter at 50 Hz was also used. The EEG signals were sampled at 1024 Hz using a data acquisition, analogue to digital card (Kethley KPCI-1800) and then stored for offline signal processing.

### EEG signal pre-processing

EEG recordings were analysed with the Fieldtrip version 11.11.15 (Donders Institute for Brain, Cognition and Behaviour, Radboud University Nijmegen, Netherlands)^30^ in Matlab 2015a (MathWorks Inc., MA, USA). For segmentation, each target stimulus onset was used to define a single trial that included 1000 ms pre-stimulus and 1000 ms post-stimulus signal. For the pre-processing, a four-step protocol was implemented utilising Fieldtrip. Initially, each one of the 61 channel signals corresponding to the 61 electrode sites was presented for each trial, and then signals for all trials were presented for each channel. Trials or channels with high impedance, EMGs, ocular blinks and trend derived contaminations were removed manually. The next step involved removal of trials and channels according to their inter-correlated variance. Finally, an Independent Component Analysis was performed to further eliminate artefacts, ECG, EMG, high impedance electrodes and alpha-wave activity. Criteria for determining these ICA components were their scalp location, time course and spectral power of average signal of each component but also spectral power from three randomly selected trials of the same component. Participants with noisy signals, intensive eye blinking and EMG were excluded from further analysis. Furthermore, participants selected for analysis had at least 80% of their trials maintained after signal pre-processing. Using these criteria we retained EEG data from 13 out of 17 subjects in the mixed design experiment and data from all 10 subjects in the block design experiment. The average number of trials per subject, per condition that were retained for the mix design are the following: Mean for Reliable-Valid = 94.6, SD = 9.9; Mean for Reliable-Invalid = 29.9, SD = 4.4; Mean for Reliable-Catch = 34.2, SD = 3.2; Mean for Unreliable-Valid = 30.7, SD = 3.2; Mean for Unreliable-Invalid = 92.5, SD = 12.3; Mean for Unreliable-Catch = 34.5, SD = 3.2. The average number of trials per subject, per condition that were retained for the block design are the following: Mean for Reliable-Valid = 85.4, SD = 10.9; Mean for Reliable-Invalid = 28.0, SD = 4.1; Mean for Reliable-Catch = 30.9, SD = 3.3; Mean for Unreliable-Valid = 30.2, SD = 3.5; Mean for Unreliable-Invalid = 89.2, SD = 7.3; Mean for Unreliable-Catch = 31, SD = 3.7.

Pre-processed EEG signals were re-referenced at the average signal of all electrodes. Baseline correction was performed using the period of 300–100 ms before the target stimulus onset in order to examine post-stimulus effects^31^. Average ERPs were then obtained for each subject and task condition and then were filtered using a low-pass filter with a 30 Hz cut-off.

### P300 ERP analysis

The mixed and block design experiments were analysed separately. All cue target trials were averaged and compared to the average catch trials for every electrode (Supplementary Fig. 1). Identification of the P300 component was based on morphological and spatiotemporal characteristics, as described in the literature^4^. An averaged waveform from electrodes CP_1_, CP_2_, CP_Z_, P_1_, P_2_ and P_Z_ best captured the P300 potential and was utilized in order to minimize the Type I errors due to multiple comparisons derived from introducing the electrode site as a factor in ANOVA^31^. The onset time for the P300 was determined as the moment when cue target trial average waveform departed significantly from the catch trial average waveform in a time window between 200 ms and 500 ms following the target. A Wilcoxon Signed rank test was used to assess significance. The resulting p-values were corrected for multiple comparisons using the False Discovery Rate correction with α = 0.05.

For every subject and every trial type (reliable valid, reliable invalid, unreliable valid, unreliable invalid) the mean P300 amplitude was calculated by averaging the ERP amplitude from 350 to 450 ms after stimulus onset. This time window was selected based on the initial cue target versus catch trial analysis to minimise contributions from previous or subsequent ERP components. The peak P300 latency was estimated by locating the time point which contained the maximum value in the 200–500 ms interval after stimulus onset.

The subject mean P300 amplitude or latency measures for each experiment (mixed and block design) were entered in a repeated measures ANOVA with cue validity and reliability as within subject factors. To test the hypotheses of target probability and cue match probability hypothesis we used the same planned comparisons as the ones used in the analysis of RT data. The General Linear Model module of STATISTICA 10.0 software (StatSoft Inc. 1984–2011) was used for statistical analysis.

To further examine the power of significant and non-significant effects of stimulus reliability and validity on P300 amplitude we used Bayesian paired samples t-tests. Bayesian analysis is able to assess how strong is the evidence for the null hypothesis (no difference between two conditions) against the alternative hypothesis (a difference between two conditions) or the opposite. We performed a two-way Bayesian t-test to show how strong the evidence was for the null hypothesis of no main effect of reliability on P300 amplitude. To estimate the reliability/validity interaction effect we subtracted the mean P300 amplitude of valid trials from the mean P300 amplitude of invalid trials for reliable cues and repeated this for unreliable cues. This was calculated for each subject. Then we performed a two-way Bayesian t-test to show how strong the evidence was for the null hypothesis of no validity/reliability interaction effect on P300 amplitude. Finally we used a one-sided Bayesian t-test to show how strong is the evidence for the alternative hypothesis against the null, that the P300 amplitude was larger for invalid trials compared to valid trials. The Bayesian analysis was performed using JASP software (Version 0.11.1; JASP Team, 2019) to estimate a Bayes factor using Bayesian Information criteria^32^. The Cauchy prior was set at the JASP default of 0.707.

## Supplementary information

Supplementary Material.
