# Supplementary material for: P300 response modulation reflects breaches of non-probabilistic expectations
Source: Sci Rep. 2020 Jun 24;10:10254. doi: 10.1038/s41598-020-67275-0 (PMC7314850; doi:10.1038/s41598-020-67275-0)
Supplement: Supplementary file 1 — Supplementary Material. [file 41598_2020_67275_MOESM1_ESM.pdf]

P300 response modulation reflects breaches of non-probabilistic expectations

Valakos D.[1,9], d'Avossa G.[2], Mylonas D[4,5], Butler J[6], Klein C[3,7,8], Smyrnis N.[1,3*]

[1]Laboratory of Cognitive Neuroscience and Sensorimotor Control, University Mental Health, Neurosciences and Precision Medicine Research Institute "COSTAS STEFANIS", Athens, Greece.

[2]Department of Psychology, Bangor University, Bangor, UK.

[3]Department of Psychiatry, National and Kapodistrian University of Athens, Medical School, Eginition Hospital, Athens, Greece.

[4]MGH/HST Athinoula A. Martinos Center for Biomedical Imaging, Massachusetts General Hospital, Boston, MA, USA.

[5]Department of Psychiatry, Harvard Medical School, Boston, MA, USA.

[6] School of Psychology, Faculty of Health and Wellbeing, University of Sunderland, Sunderland, UK.

[7]Department of Child and Adolescent Psychiatry, University of Freiburg, Germany.

[8]Department of Child and Adolescent Psychiatry, Medical Faculty, University of Cologne, Germany.

[9]Faculty of Biology, National and Kapodistrian University of Athens, Panepistimioupoli Zografou, Athens, Greece.

*Correspondence: Nikolaos Smyrnis, Psychiatry Department, National and Kapodistrian University of Athens, Medical School, Eginition Hospital, 72 V. Sofias Ave., Athens, GR 11527, Greece.

Email: smyrnis@med.uoa.gr.

Supplementary Material

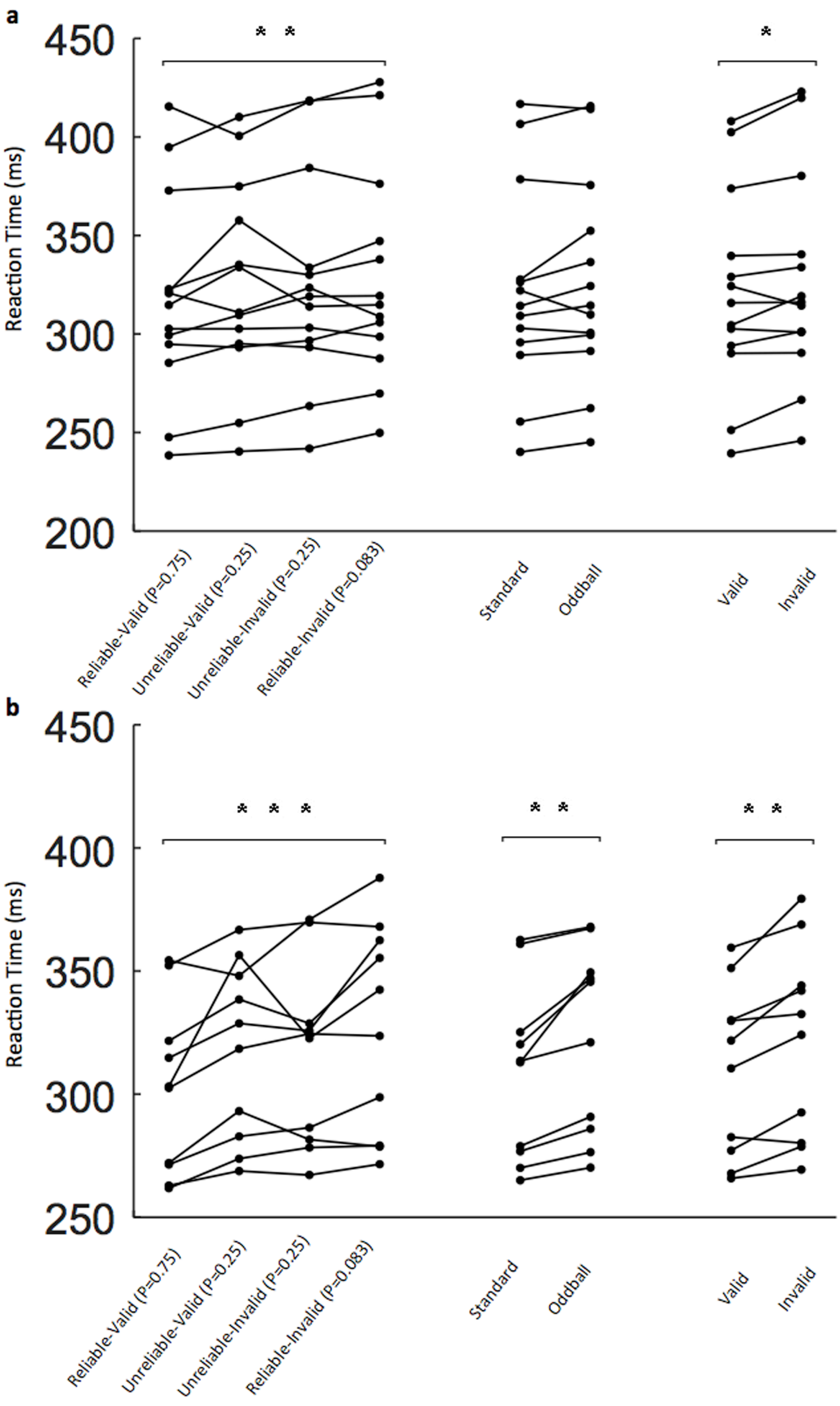

Supplementary Figure 1: Single-subject data for the mix (a) and the block (b) experiments depicting the Reaction Time for the target location probability, that cue match probability and the target validity hypotheses. *: $p<0.05$, **: $p<0.01$, ***: $p<0.001$.

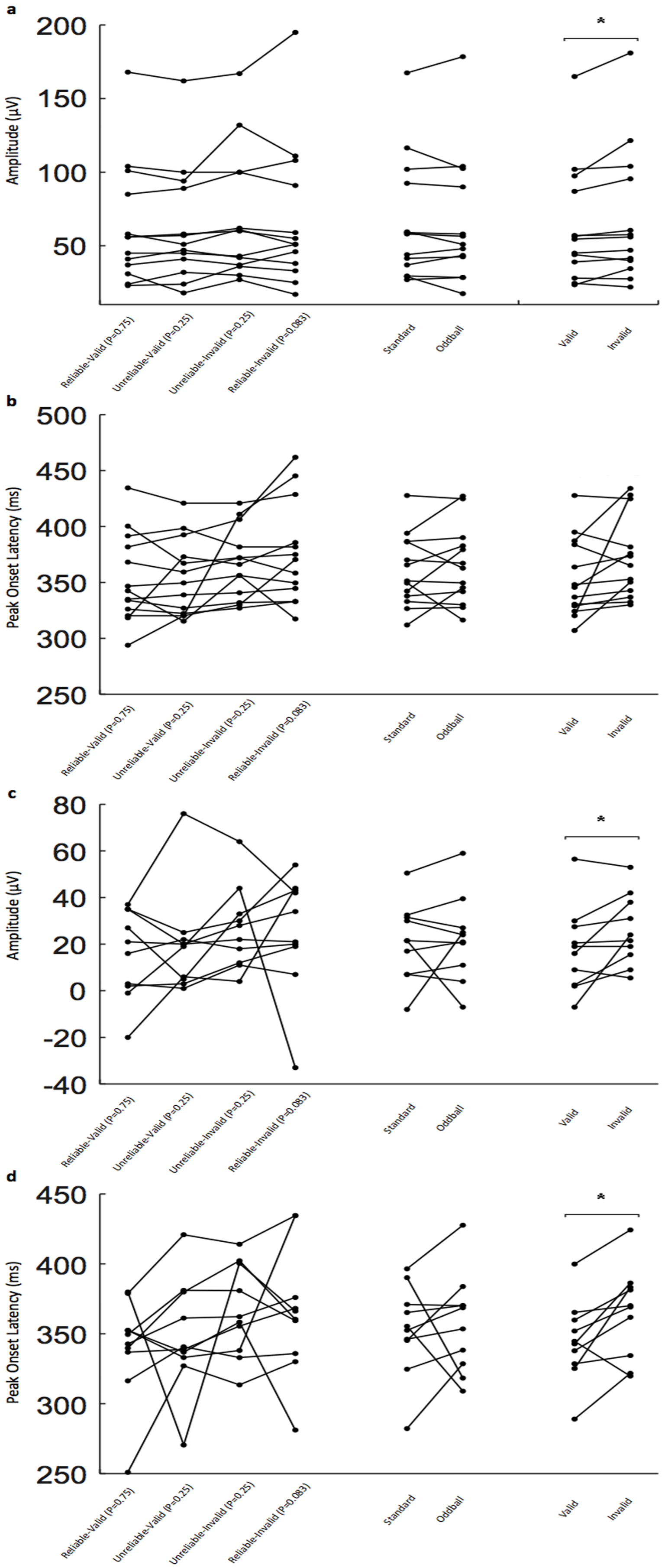
a
Amplitude (µV)
200
150
100
50
*
Reliable-Valid (P=0.75)
Unreliable-Valid (P=0.25)
Unreliable-Invalid (P=0.25)
Reliable-Invalid (P=0.083)
Standard
Oddball
Valid
Invalid
b
Peak Onset Latency (ms)
500
450
400
350
300
250
Reliable-Valid (P=0.75)
Unreliable-Valid (P=0.25)
Unreliable-Invalid (P=0.25)
Reliable-Invalid (P=0.083)
Standard
Oddball
Valid
Invalid
c
Amplitude (µV)
80
60
40
20
0
-20
-40
*
Reliable-Valid (P=0.75)
Unreliable-Valid (P=0.25)
Unreliable-Invalid (P=0.25)
Reliable-Invalid (P=0.083)
Standard
Oddball
Valid
Invalid
d
Peak Onset Latency (ms)
450
400
350
300
250
*
Reliable-Valid (P=0.75)
Unreliable-Valid (P=0.25)
Unreliable-Invalid (P=0.25)
Reliable-Invalid (P=0.083)
Standard
Oddball
Valid
Invalid

Supplementary Figure 2: Single-subject data depicting mean P300 amplitude and peak onset latency for target location probability, cue match probability and target validity hypotheses in the mixed design (**a** and **b** respectively) and in the blocked design (**c** and **d** respectively). *: $p<0.05$.

Stimulus	Cue	Validity	Target Validity Probability	Target Location Probability	Mapping
	Reliable	Valid	36/48 (75%)	36/48 (75%)	
	Reliable	Invalid	12/48 (25%)	4/48 (8,3%)	
	Reliable	Catch	13	13	
	Unreliable	Valid	12/48 (25%)	12/48 (25%)	
	Unreliable	Invalid	36/48 (75%)	12/48 (25%)	
	Unreliable	Catch	13	13	

Supplementary Table 1: Representation of the stimuli presented in the task and classification of them according to the three different hypotheses. The trial is classified as valid if the cue predicts correctly the location of the target, and as invalid if the prediction is wrong. Target validity probability is defined by the nature of the cue. Reliable (blue) cues suggest that there is a 75% probability that the target will appear at the indicated location (standard condition) and 25% that it will appear at a different location (oddball condition). Unreliable (red) cues suggest that the probability that the target appears at the indicated

location is 25% (oddball condition), while the probability that the target will appear at a different location from the indicated one is 75% (standard condition). Target location probability is defined by the actual location of the target. In case of a reliable cue trial, there is a probability of 75% that the target will appear at the indicated location and each one of the other three locations share a probability of 8.3%, whereas in case of an unreliable cue trial, the probability that the target will appear at the indicated location equals to 25%, which is also the same for all the other three locations. Catch trials are excluded from the probability calculation since they do not elicit a sensorimotor response. The final column specifies the mapping of each condition which is followed throughout the manuscript.
